# Kinin B_1 _receptors mediate depression-like behavior response in stressed mice treated with systemic *E. coli *lipopolysaccharide

**DOI:** 10.1186/1742-2094-7-98

**Published:** 2010-12-31

**Authors:** Alice F Viana, Izaque S Maciel, Fabiana N Dornelles, Claudia P Figueiredo, Jarbas M Siqueira, Maria M Campos, João B Calixto

**Affiliations:** 1 Pharmacology Department, Universidade Federal de Santa Catarina, Florianópolis, SC, Brazil; 2 Faculty of Pharmacy, Pontifícia Universidade Católica do Rio Grande do Sul, Porto Alegre, RS, Brazil; 3 Faculty of Dentistry, Pontifícia Universidade Católica do Rio Grande do Sul, Porto Alegre, RS, Brazil; 4 Institute of Toxicology, Pontifícia Universidade Católica do Rio Grande do Sul, Porto Alegre, RS, Brazil

## Abstract

**Background:**

Kinin B_1 _receptors are inducible molecules up-regulated after inflammatory stimuli. This study evaluated the relevance of kinin B_1 _receptors in a mouse depression behavior model.

**Methods:**

Mice were exposed to a 5-min swimming session, and 30 min later they were injected with *E. coli *lipopolysaccharide (LPS). Depression-like behavior was assessed by determining immobility time in a tail suspension test. Different brain structures were collected for molecular and immunohistochemical studies. Anhedonia was assessed by means of a sucrose intake test.

**Results:**

Our protocol elicited an increase in depression-like behavior in CF1 mice, as assessed by the tail-suspension test, at 24 h. This behavior was significantly reduced by treatment with the selective B_1 _receptor antagonists R-715 and SSR240612. Administration of SSR240612 also prevented an increase in number of activated microglial cells in mouse hippocampus, but did not affect a reduction in expression of mRNA for brain-derived neurotrophic factor. The increased immobility time following LPS treatment was preceded by an enhancement of hippocampal and cortical B_1 _receptor mRNA expression (which were maximal at 1 h), and a marked production of TNFα in serum, brain and cerebrospinal fluid (between 1 and 6 h). The depression-like behavior was virtually abolished in TNF*α *p55 receptor-knockout mice, and increased B_1 _receptor mRNA expression was completely absent in this mouse strain. Furthermore, treatment with SSR240612 was also effective in preventing anhedonia in LPS-treated mice, as assessed using a sucrose preference test.

**Conclusion:**

Our data show, for the first time, involvement of kinin B_1 _receptors in depressive behavioral responses, in a process likely associated with microglial activation and TNFα production. Thus, selective and orally active B_1 _receptor antagonists might well represent promising pharmacological tools for depression therapy.

## Background

Chronic depression represents a public health worldwide problem. Despite the existence of several drugs for depression treatment, these medicines have many significant adverse effects, and many patients do not display satisfactory responses to the current therapeutic arsenal [1]. The etiology of depression is incompletely understood, and this precludes development of more effective drugs. Compelling literature data suggests a crosstalk between immunological changes and major depression [2,3]. It has been demonstrated that systemic administration of pro-inflammatory cytokines or some bacterial products to rodents elicits a condition described as sickness behavior, characterized by decreased food consumption and locomotor activity, social isolation, and changes in the circadian cycle, which is followed by depressive behavior [3-5].

Toll-like receptors (TLR) are recognition units that distinguish microbial structures. Gram-negative bacterial lipopolysaccharide (LPS) commonly signals through TLR4, leading to activation of several intracellular pathways [6]. It has been demonstrated that depression-like behavior induced by LPS in rodents is dependent on cytokine production; importantly, depressed patients display elevated cytokine plasma levels [2,3]. Furthermore, it has been recently shown that TLR activation following infection can induce systemic inflammation, accompanied by signs of brain-controlled illness in rats [7,8].

Kinins are a group of peptides that are rapidly generated in response to several stimuli [9]. Once released, kinins activate two G protein-coupled receptors, called B_1 _and B_2_. B_2 _receptors are constitutively expressed throughout several tissues, whereas B_1 _receptors are not commonly expressed under normal conditions, although they are rapidly upregulated after infection, trauma, or by certain cytokines [10-13]. Of relevance, a series of previous publications has demonstrated an important role for TNFα in the up-regulation of kinin B_1 _receptors [14-17]. Therefore, B_1 _receptors are likely induced under certain pathological conditions, being involved in several chronic inflammatory and pain processes [9,10].

Previous studies have demonstrated that LPS from either *E. coli *or *P. gingivalis *can induce a marked up-regulation of kinin B_1 _receptors in animal models of peripheral inflammation, via cytokine production [14,15,18]. Of relevance, recent studies also suggest that kinin B_1 _receptors have also been implicated in some diseases involving the central nervous system (CNS), such as epilepsy, Alzheimer's disease and neuropathic pain [19-21]. Taking into account the above mentioned data, the present study was designed to test the hypothesis that kinin B_1 _receptors might be implicated in depression-like behavioral changes elicited by systemic administration of *E. coli *LPS, in mice submitted to a previous stressing forced swimming session. This experimental protocol was based on the concept that internal and external stressors are able to interact, culminating in a general illness state, which causes an allostatic overload [2,22,23]. Efforts have also been made to define some of the mechanisms responsible for B_1 _receptor induction in the context of LPS-treated depressed animals by using biochemical and molecular techniques, such as flow cytometry, ELISA and real-time PCR. We have also aimed at determining whether antagonism of kinin B_1 _receptors could modulate glial activity, throughout immunohistochemical studies. Finally, we further investigated the role played by TNFα in the depressive-like behavior in our experimental paradigm, by using TNF*α *p55 receptor-deficient mice.

## Methods

### Materials

The following drugs and reagents were used: imipramine, LPS from *E. coli *serotype 0111:B4, aprotinin A, benzethonium chloride, EDTA, HTAB, hydrogen peroxide, PMSF, TMB and Tween-20 (all from Sigma Chemical Company St. Louis, U.S.A); R-715, kindly provided by Dr. Domenico Regoli (University of Sherbrooke, Sherbooke, Quebec, Canada); SSR240612 was kindly provided by Sanofi-Synthelabo Recherche (Montpellier, France). FR173657 was donated by Fournier Laboratories (Dijon, France). The stock solutions of the drugs were prepared in PBS in siliconized plastic tubes, maintained at -18°C, and diluted to the desired concentration just before use.

### Animals

Male CF1 and C57/BL6 wild-type, or TNFα p55 receptor knockout mice (25 to 30 g) were used in this study. Animals were housed under conditions of optimum light, temperature and humidity (12 h light-dark cycle, 22 ± 1°C, under 60 to 80% humidity), with food and water provided *ad libitum*. CF1 mice were obtained from the central animal house of the Universidade Federal de Pelotas (UFPEL, Brazil). C57/BL6 wild-type, or TNFα p55 receptor knockout mice were supplied by the Universidade Federal de Minas Gerais (UFMG, Belo Horizonte, Brazil). All experiments were performed between 08:00 AM and 08:00 PM. Experiments were conducted in accordance with current guidelines for the care of laboratory animals and ethical guidelines for the investigation of experimental pain in conscious animals laid down by Zimmermann (1983) [24]. All the experimental procedures were approved by the Animal Ethics Committees of Universidade Federal de Santa Catarina (SC) and Pontifícia Universidade Católica do Rio Grande do Sul (RS).

### Induction of depressive-like behavior

As a pre-stressful stimulus, the animals were subjected to forced swimming for 5 min, in a water temperature of 23 ± 1°C. Subsequently, they were injected with LPS from *E. coli *(serotype 0111:B4) at the doses of 450 mg/kg (CF1 mice) or 1000 μg/kg (C57/BL6 wild-type, or TNFα p55 receptor knockout mice), by i.p. route. The control groups received saline (0.9% NaCl solution, 10 ml/kg, i.p.). The protocol of forced swimming and the selected doses of LPS were determined on the basis of pilot experiments (not shown).

The animals were assessed in behavioral paradigms at 6, 24 or 48 h time points after LPS administration, depending on the experimental protocol. All the behavioral parameters were evaluated by trained experimenters blind to the treatment groups. Separate groups of mice were euthanized at different time-points after LPS injection, in order to perform biochemical, molecular biology and immunohistochemical assays, as described in the next sections.

### Protocols of treatment with kinin antagonists

To verify the involvement of kinin receptors in the behavioral changes elicited by LPS from *E. coli*, the animals were treated with one of the following drugs before behavioral tests: the selective antagonists of kinin B_1 _receptor SSR240612 (5 mg/kg, i.p., 30 min; or 10 mg/kg, p.o., 1 h) or R-715 (0.5 mg/kg, i.p., 30 min), or the selective kinin B_2 _receptor antagonist FR173657 (30 mg/kg, i.p., 30 min). The classical tricyclic antidepressant imipramine (10 mg/kg, i.p., 30 min) was used as a positive control drug. Control animals received saline solution (0.9% NaCl solution, 10 ml/kg), at the corresponding schedules of treatment used for the antagonists. For molecular biology and immunohistochemical studies, animals were treated with SSR240612 (5 mg/kg, i.p.), 30 min before LPS, and were sacrificed at defined time-points as described in the next sections. The doses of kinin antagonists and imipramine were determined on the basis of previous publications [25-27].

### Tail-suspension test

To assess the depression-like behavior following the 5-min forced swimming session in animals treated with LPS from *E. coli*, we employed a tail-suspension model according to the methodology originally described by Steru *et al*. (1985) [28]. At different time intervals after LPS treatment (6, 24 or 48 h), the animals were suspended 50 cm above the floor by means of an adhesive tape, placed approximately 1 cm from the tip of the tail. The time during which mice remained immobile was quantified (in s) over a period of 6 min.

### Sucrose consumption test

Anhedonia represents decreased sensation of pleasure, which can be evaluated in mice by measuring reduction of sucrose intake. The protocol used in our study was adapted from the method described by Strakaliva et al, 2004 [29]. For three days, mice received 1% sucrose solution, and they were subjected to forced swimming, twice a day, for a total of six sessions (temperature water 16 to 19°C, for 5 min), as a pre-stressful stimuli. After the last swimming session, mice were treated with LPS from *E. coli *(450 μg/kg, i.p), followed by a 24-h period of food and water deprivation, according to the original protocol [29]. Sucrose intake was assessed for 12 h, where mice had free access to two bottles, one with 1% sucrose solution and another with tap water. The weight differences of bottles were used to calculate the consumption of sucrose with the following formula: % sucrose intake = [sucrose intake (g)] × 100/[sucrose intake (g) + tap water (g)]. The animals were treated with the selective kinin B_1 _receptor antagonist SSR240612 (10 mg/kg, p.o., 1 h) or the antidepressant imipramine (10 mg/kg, i.p., 30 min), both administered before LPS injection. Control animals received saline solution, at same schedule of treatment.

### Open-field test

To analyze the effects of swimming session plus LPS treatment on the locomotor activity, the animals were evaluated in an open-field test [30], 6 or 24 h after endotoxin administration. The experiments were conducted in a sound-attenuated room, under low-intensity light. Mice were individually placed in the centre of an acrylic box (40 × 60 × 50 cm), with the floor divided into 9 squares. The number of squares crossed with the four paws was registered over a period of 6 min.

### Body temperature assessment

Mouse colonic temperature was recorded using a commercially available thermometer (Pro-check^®^), which was dipped in Vaseline and inserted about 0.5 cm into a gently hand-restrained mouse. After recording the initial colonic temperature (t = 0), the body temperature of mice was evaluated 6 or 24 h after LPS injection.

### Real-time PCR quantification of kinin B_1 _receptor and BDNF mRNA

Mouse hippocampi and cortex were collected using a special apparatus, after euthanasia by decapitation, at 1, 3, 6 and 24 h following LPS injection. Total RNA was extracted using Trizol reagent (Invitrogen), according to the manufacturer's instructions. The concentration of total RNA was determined by measuring the absorbance at 260 nm. In order to obtain the reverse transcript (cDNA), 2 μg of total RNA were reverse transcribed using oligo(dT) as a primer (0.05 μg), 50 U of reverse transcriptase (Promega), dNTP (144 μM; Promega), reaction buffer [10 mM dithiothreitol (DTT), 3 mM MgCl2, 75 mM KCl, and 50 mM Tris-HCl, pH 8.3], and 2 U of RNAsin Plus (Promega), in a final volume of 12.5 μl. The cDNA was obtained after incubation of the samples for 5 min at 70°C, 4°C for 5 min, 37°C for 60 min, 70°C for 5 min, and 4°C for 5 min.

B_1 _receptor and BDNF mRNA expression was carried out through fluorescence-based real-time PCR. To this end, approximately 100 ng of cDNA were amplified in duplicates using TaqMan-based chemistry with specific primers and FAM-labeled probes for mouse BDNF (cat# Mm00432069_m1), B_1 _receptor (cat# Mm00432059_s1) and glyceraldehyde-3-phosphate dehydrogenase (GAPDH; cat# Mm03302249_g1) as the endogenous control for normalization. Amplifications were carried out in a Thermalcycler (StepOne Plus, Applied Biosystems) for 50 cycles; the fluorescence was collected at each amplification cycle and the data analyzed using the 2^-ΔΔCt ^method for relative quantification. Expression of the target genes was calibrated against conditions found in naive animals, i.e., non-treated mice.

### Immunohistochemical studies

Brain samples were collected 24 h following LPS treatment, and fixed in a phosphate buffered saline (PBS) solution containing 4% paraformaldehyde for 24 h at room temperature. Subsequently, the samples were submitted to standard histological proceeding in order to be embedded in paraffin. Hippocampus sections were cut approximately at the level of 3 mm from bregma [2,31]. To determine glial activity, immunohistochemistry was carried out on paraffin tissue sections using CD68 antibody (1:150; Cell Signaling Technology, Beverly, MA, USA). Following quenching of endogenous peroxidase with 1.5% hydrogen peroxide in methanol for 20 min, high temperature antigen retrieval was performed by immersion of the slides in a water bath at 95 to 98°C in 10 mM trisodium citrate buffer pH 6.0, for 45 min. After overnight incubation at 4°C with the primary antibody, the slides were washed with PBS, incubated with the appropriate biotinylated secondary antibody (Dako Cytomation), and then processed using the Streptavidin-HRP reagent (Dako Cytomation), according to the manufacturer's instructions. Sections were developed with DAB (3,3'-diaminobenzidine) (Dako Cytomation) in chromogen solution and counterstained with Harris's hematoxylin. Control and experimental tissues were placed on the same slide and processed under the same conditions.

Images were acquired by Sight DS-5M-L1 digital camera connected to a light microscope Eclipse - 80i (Nikon). Settings for image acquisition were identical for control and experimental tissues. For each mouse, the number of CD-68 positive cells was counted in four different fields of CA1, CA2, CA3 and dentate girus (DG) hippocampal sub regions, using 40X magnification.

### Measurement of TNFα levels by ELISA

The animals were submitted to forced swimming and treated with LPS from *E. coli*, as described before. Subsequently, they were euthanized by decapitation at distinct intervals of time following LPS (1, 3, 6, 12 and 24 h). TNFα levels were determined in serum or in whole brain by means of a standard sandwich ELISA technique, according to the recommendations of the supplier (R&D Systems, USA). For the brain assays, the tissues were removed and placed in a PBS solution containing 0.05% Tween 20, 0.1 mM PMSF, 0.1 mM benzamethonium chloride, 10 mM EDTA, 2 μg/ml aprotinin A, and 0.5% BSA. The mouse brains were homogenized and then centrifuged at 3,000× g for 10 min, and the supernatant was employed for ELISA analysis.

### 2.10. Preparation of samples for flow cytometry

Cerebral spinal fluid (CSF) samples were taken from the cisterna magna using a method adapted from Liu and Duff [32]. Mice were submitted to forced swimming and treated with LPS from *E. coli *(450 μg/kg, i.p.), as described before. One, three or twenty four h after treatment, mice were anesthetized with a mixture of ketamine (100 mg/kg; i.p.) and xylazine (10 mg/kg; i.p.), and placed on stereotaxic instrument. The head was positioned in a 135° angle with the body, and a sagittal incision of the skin was made inferior to the occiput. To aspirate the CSF, the metal part of an insulin needle was adapted at one end of a polyethylene 50 tubing, and at the other end a 50 μl Hamilton syringe was attached. The needle was attached to the bottom end of the manipulator bar, and 3 mm of it was inserted into the cisterna magna. The CSF collected was transferred to a 0.5 ml Eppendorf tube, and frozen immediately on dry ice and then kept into -80°C until use. CSF from naïve animals was used as control.

### Flow cytometer acquisition and data analysis

The BD Cytometric Bead Array (CBA) was used to quantitatively measure cytokines in the CSF. The CBA Mouse Inflammation Kit^® ^(BD Bioscience, San Jose, CA) was used according to the manufacturer's instructions to simultaneously detect interleukin-6 (IL-6), interleukin-10 (IL-10), interferon-γ (IFN-γ), tumor necrosis factor alpha (TNFα), and interleukin-12p70 (IL-12p70). Flow cytometer readings were performed using FACSCanto II red laser in a medium range of 633 nm. Data were acquired using FACSDiva software and analyzed using FCAP Array software (all from BD Bioscience). The captured cytokines were detected via direct immunoassay using six different antibodies coupled to phycoerythrin (PE). Standard curves were generated for each cytokine using the mixed cytokine beads standard provided by the kit. The concentration of the standards ranged from 20 to 5000 pg/ml. Five standard curves were plotted such as: cytokine concentration X mean fluorescence intensity (MFI), using a four-parameter logistic curve fitting model. The concentrations of each CSF cytokine were determined from these four-parameter equations using the MFI value of each cytokine. If a sample had a cytokine concentration below the detection limit for the assay, a value of 0 was assigned for that concentration.

### Statistical analysis

For the behavioral parameters and Elisa protocols, the results are presented as the mean ± SEM of 6 to 8 animals. For the real-time PCR, flow cytometry and immunohistochemical experiments, the results are given as the mean ± SEM of 4 independent experiments, performed in triplicate. The statistical comparison between these values was performed by one-way analysis of variance followed by Newman-Keuls post hoc test. P values less than 0.05 (*p *< 0.05) were considered as indicative of significance.

## Results

### General behavioral data

**Figure 1:**
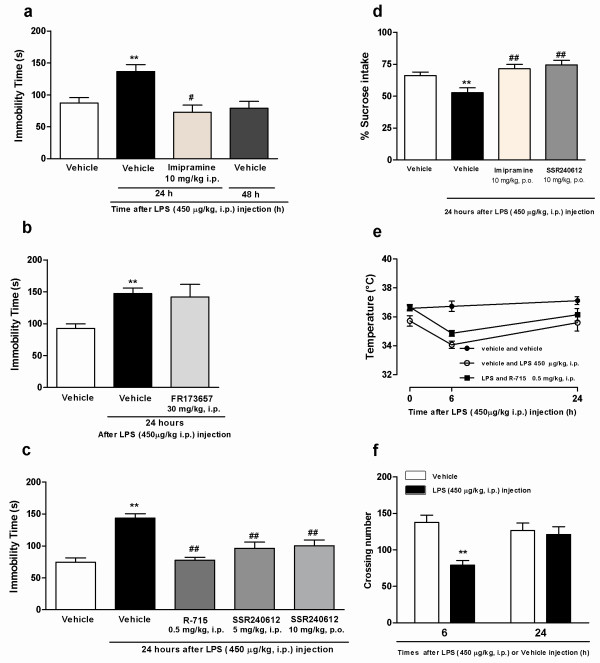
**Effects of acute inflammation on sickness and depression-like behavior in mice; involvement of kinin B_1 _receptor**. Effect of systemic administration of *E. coli *LPS (450 μg/kg, i.p., 24 h and 48 h beforehand) on immobility time in mice submitted to a previous 5-min swimming session in the tail suspension test (a). Influence of imipramine (10 mg/kg i.p.), given 30 min before, on the increased immobility time in pre-stressed animals treated with *E. coli *LPS. (b) Effect of the selective kinin B_2 _receptor antagonist FR173657 (5 mg/kg, i.p., 30 min). (c) Effect of the selective kinin B_1 _receptor antagonists R-715 (0.5 mg/kg, i.p., 30 min) or SSR240612 (5 mg/kg, i.p., 30 min or 10 mg/kg, p.o., 1 h). (d) Effect of systemic administration of *E. coli *LPS (450 μg/kg, i.p.) after three days of forced swimming on sucrose intake; influence of imipramine (10 mg/kg, i.p., 30 min) or SSR240612 (10 mg/kg p.o., 1 h) treatment. (e) Effect of *E. coli *LPS (450 μg/kg, i.p., 6 h and 24 h) on body temperature, in mice submitted to a previous 5-min swimming session; influence of treatment with R-715 (0.5 mg/kg, i.p., 30 min). Effect of systemic administration of *E. coli *LPS (450 μg/kg, i.p., 6 h and 24 h) on locomotor activity in mice submitted to a previous 5-min swimming session (f). Each column or point represents the mean of 6-8 animals and vertical lines show the SEM. **P < 0.01 compared to vehicle and ^#^P < 0.05, ^##^P < 0.01 compared LPS-treated mice.

The results depicted in Figure 1a demonstrate that systemic administration of *E. coli *LPS (450 μg/kg, i.p.), following a 5-min forced swimming session, elicited time-related depression-like behavior in mice, according to assessment in the tail-suspension paradigm. The increase of immobility time in animals pre-treated with LPS was statistically significant at 24 h (*P *< 0.01), but not at 6 h (P > 0.05; results not shown), returning to control levels at 48 h (*P *< 0.05), in comparison to saline-treated mice. As could be expected, treatment with the classical antidepressant drug imipramine (10 mg/kg, i.p.), given 30 min before the behavioral assessment, produced a marked reduction of the immobility time (47 ± 16% of reduction; Figure 1a). Of interest, i.p. treatment with the selective B_1 _receptor antagonists R-715 (0.5 mg/kg, 30 min) or SSR240612 (5 mg/kg, i.p., 30 min), or even oral administration of SSR240612 (10 mg/kg, 1 h), caused a significant inhibition of depression-like behavior induced by LPS (Figure 1c). The percentages of inhibition for animals treated with B_1 _receptor antagonists were: 46 ± 6%, 33 ± 7% and 30 ± 6%, respectively. On the other hand, the selective kinin B_2 _receptor antagonist FR173657 (30 mg/kg, i.p., 30 min) failed to significantly alter the immobility time in our model (*P *> 0.05; Figure 1b). Injection of LPS after three days of forced swimming in Swiss mice led to a significant decrease of sucrose consumption (*P *< 0.05). The decreased sucrose intake was notably prevented by pre-treating animals with the antidepressant imipramine (10 mg/kg, i.p., 30 min) or with the B_1 _receptor antagonist SSR240612 (10 mg/kg, p.o, 1 h) (*P *> 0.05; Figure 1d).

It is well known that LPS treatment evokes some classical CNS-associated changes in rodents [3-5]. Confirming literature data, our results demonstrate that *E. coli *LPS administration in mice previously submitted to forced swimming, is able to cause a marked reduction of body temperature (Figure 1e), and of locomotor activity when assessed in the open-field arena. The locomotor activity was significantly reduced at 6 h after LPS (*P *< 0.05), returning to the control levels at 24 h (Figure 1f). Body temperature was found significantly reduced at 6 h (*P *< 0.01), but not at 24 h after LPS (*P *> 0.05). Remarkably, the administration of R-715 (0.5 mg/kg, i.p.), 30 min before the test, did not display any significant effect on this parameter as assessed at 6 h (Figure 1e). Considering the oral bioavailability of SSR240612, the next experiments were carried out using only this antagonist.

### Biochemical and molecular approaches

**Figure 2:**
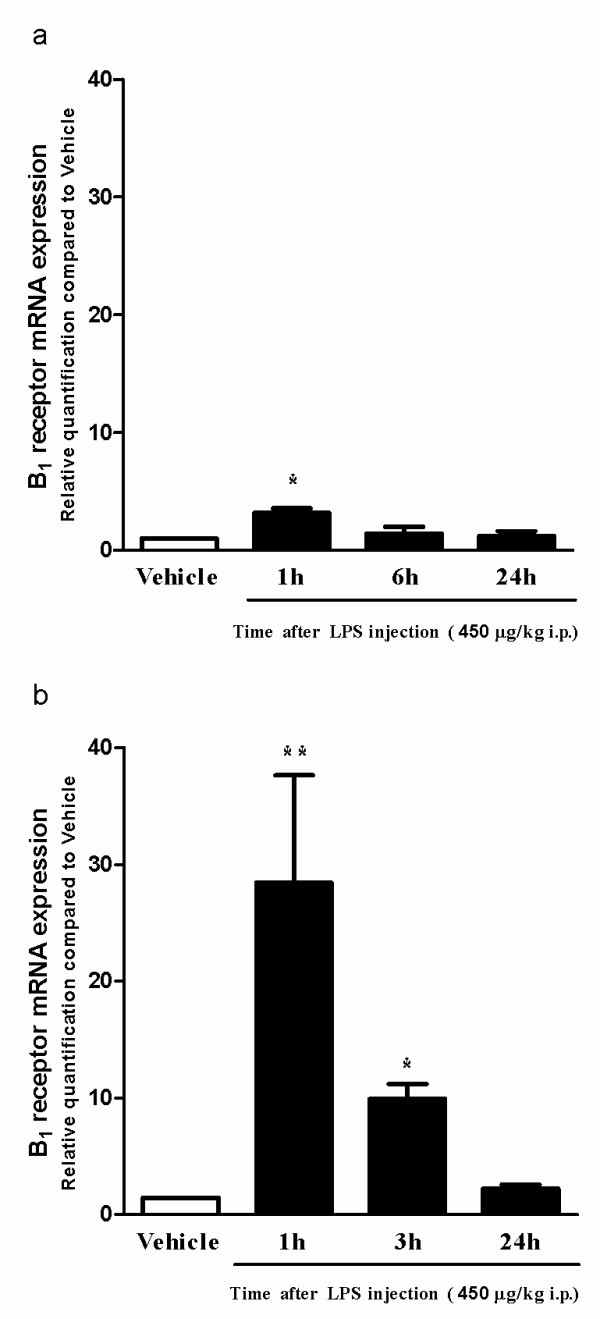
**Expression of B_1 _receptor mRNA in hippocampus and cortex of mice after acute inflammation**. Time-related effects of a 5-min forced swimming session plus *E. coli *LPS treatment (450 μg/kg, i.p.) on B_1 _receptor mRNA expression in the hippocampus (a) and cortex (b) of mice. Each column represents the mean of 4 animals and vertical lines show the SEM. *P < 0.05 and **P < 0.01 compared to vehicle-treated mice.

We wondered whether the effects of selective kinin B_1 _receptor antagonists might be related to changes of B_1 _receptor expression in CNS structures. Therefore, B_1 _receptor mRNA expression was measured in the hippocampus and frontal cortex of mice submitted to a session of forced swimming, followed by LPS administration (450 μg/kg, i.p.). Quantitative real-time experiments revealed a time-dependent, marked increase in B_1 _receptor mRNA levels in hippocampus, which peaked at 1 h (about 2.5-fold increase) (Figure 2a) while in the cortex this increase was almost 40 fold 1 h after LPS treatment (Figure 2b).

**Figure 3:**
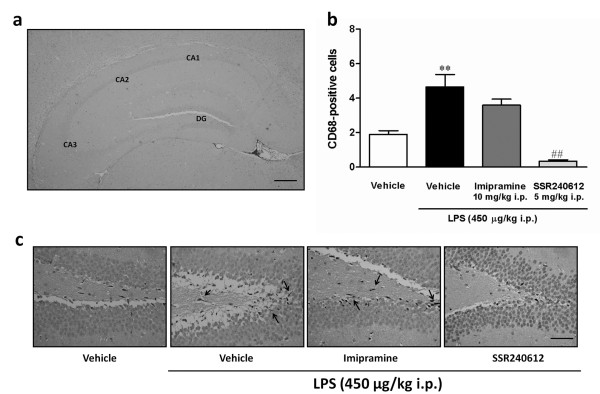
**Microglial activation in the mouse hippocampus following acute inflammation; effect of B_1 _receptor antagonism**. Effect of systemic administration of *E. coli *LPS (450 μg/kg, i.p., 24 h) on microglial activation (CD68-positivity) in the hippocampus of mice submitted to a previous 5-min forced swimming session. Mice were treated with imipramine (10 mg/kg i.p.) or SSR240612 (5 mg/kg, i.p.), given 30 min beforehand. (a) Representative image of CA1, CA2, CA3 and dentate gyrus (DG) subregions of hippocampus (Scale bar = 200 μm, original magnification, × 40). (b) Graphic representation on the average number of CD68-positive cells per field, determined in the CA1, CA2, CA3, and DG subregions of the hippocampus. (c) Representative images of CD68 immunohistochemistry in the DG subregion of hippocampus (Scale bar = 50 μm, original magnification, × 400) from pre-stressed animals treated with *E. coli *LPS, following imipramine (10 mg/kg i.p.), SSR240612 (5 mg/kg, i.p., 30 min or 10 mg/kg, p.o., 1 h), or saline 30 min before. LPS treatment showed an increase in the number of CD68-positive cells (c, arrows) in the hipocamppus, and SSR240612 (5 mg/kg, i.p., 30 min or 10 mg/kg, p.o., 1 h), was able to reduce this increase (c). Each column represents the mean of 4 animals and the vertical lines show the SEM. *P < 0.05, **P < 0.01 and ***P < 0.001 compared to vehicle-treated mice.

**Figure 4:**
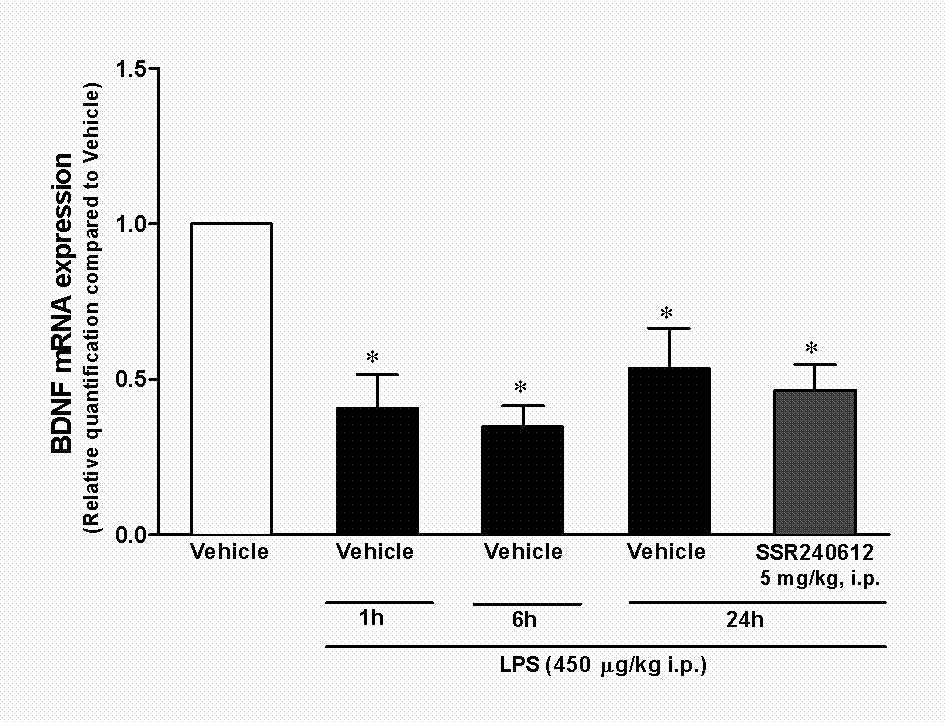
**BDNF mRNA expression in hippocampus and cortex of mice after acute inflammation; effects of B_1 _receptor antagonism**. Effect of systemic administration of *E. coli *LPS (450 μg/kg, i.p., 6 h and 24 h) on BDNF mRNA expression in the hippocampus of mice submitted to a previous 5-min forced swimming session, according to assessment by real-time PCR. Influence of treatment with SSR240612 (5 mg/kg, i.p.), given 30 min beforehand. Each column represents the mean of 4 animals and vertical lines show the SEM. *P < 0.05 compared to vehicle-treated mice.

As observed in Figure 3, forced swimming followed by LPS treatment produced a marked increase in CD68 immunoreactivity in mouse hippocampus, indicative of increased glial cell activation. Of relevance, CD68 labeling was almost completely inhibited in animals pre-treated with the selective kinin B_1 _receptor antagonist SSR240612 (5 mg/kg, i.p.), but not by the antidepressant drug imipramine (10 mg/kg, i.p.), both given 30 min before LPS. These findings suggest that different mechanisms appear to mediate imipramine and kinin B_1 _receptor antagonist antidepressant-like effects. It has been described that depression is associated with decreased expression of neurotrophic factors, such as BDNF [33-35]. In our study, real-time PCR experiments revealed a marked decrease in BDNF expression in hippocampus of mice submitted to our protocol of depression, when assessed at both the 6- and the 24-h time-points (an approximate 50% reduction). However, this parameter was not significantly affected by pre-treating animals with the kinin B_1 _receptor antagonist SSR240612 (5 mg/kg, i.p., 30 min) (Figure 4).

**Figure 5:**
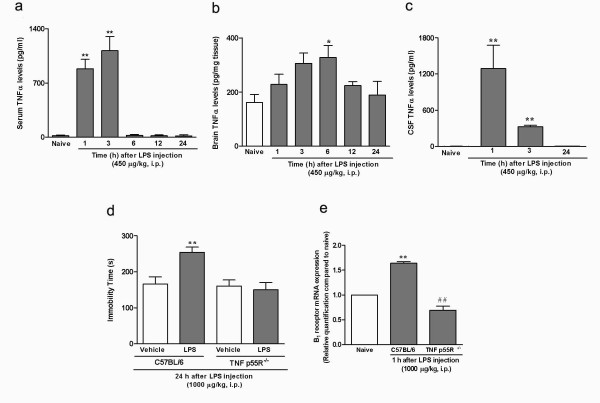
**TNFα levels in serum and brain of mice following acute inflammation; relevance of TNFα p55 receptor for B_1 _receptor mRNA expression**. Time-related effects of treatment with LPS (450 μg/kg, i.p.) on TNFα levels in serum (a), whole brain (b) or CSF (c) of CF1 mice, submitted to a previous 5-min forced swimming session, according to assessment by ELISA sandwich (a, b) or CBA (c) assays. (d) Effect of systemic administration of *E. coli *LPS (1000 μg/kg i.p., 24 h) on immobility time, in C57/BL6 or TNF*α *p55 receptor knockout mice, as assessed in the tail suspension test. (e) Evaluation of B_1 _receptor mRNA expression in C57/BL6 or TNF*α *p55 receptor knockout mice that had been previously submitted to a 5-min swimming section, and were pre-treated with *E. coli *LPS (1000 μg/kg i.p., 1 h). Each column represents the mean of 6-8 animals and vertical line show the SEM. *P < 0.05, **P < 0.01 compared to naive or vehicle-treated mice and ^##^P < 0.01 compared to C57BL/6 mice.

Next, in order to correlate the changes elicited by LPS with cytokine production, we assessed levels of TNFα in whole brain or mouse serum. The animals were submitted to a forced swimming session and treated with *E. coli *LPS, as described above. This series of results showed the occurrence of a marked, time-dependent increase in TNFα production, either in brain or in serum of LPS-treated mice. In serum, TNFα levels peaked between 1 and 3 h (an approximate 90-fold increase), returning to basal levels 6 h following LPS administration (Figure 5a). The increase of TNFα in the brain was maximal between 3 and 6 h after LPS (about 1.8-fold augmentation), being reduced to control values after 12 h (Figure 5b). We also analyzed cytokine content in CSF from LPS-treated mice, using flow cytometry analysis. The cytokines IL-6, IL-10, IFN-γ were below detection levels, while the concentration of IL-12 was similar to that observed in naïve animals (data not shown). Of interest, CSF TNFα levels peaked 1 h after LPS injection and remained elevated until 3 h later (about 340 and 90-fold increase, respectively) (Figure 5c).

Since previous literature data have shown a close interrelation between TNFα and B_1 _receptor upregulation [10,14], we further investigated the role played by TNFα in the depression-like behavior observed in our experimental paradigm. For that purpose, we employed TNF*α *p55 receptor-deficient mice. As TNF*α *p55 receptor knockout animals used in the present study are C57/BL6 inbred, we employed this mouse strain in this part of the study. In C57/BL6 mice, a dose of 1000 μg/kg of *E. coli *LPS was necessary to evoke a significant increase in immobility time in the tail-suspension test, comparable to that obtained with a 450 μg/kg dose in CF1 mice (about 1.5-fold in relation to saline-treated animals). Strikingly, the depression-like behavior caused by forced swimming plus LPS treatment (1000 μg/kg, i.p.) was virtually abolished in mice with genetic deletion of TNF*α *p55 receptors (Figure 5d). Of note, the increase of B_1 _receptor mRNA expression in mice submitted to forced swimming plus LPS treatment was completely absent in TNF*α *p55 receptor knockout animals (Figure 5e).

## Discussion

During the last few years, several advances have been achieved in our understanding of the genetic, biochemical and immunological changes implicated in depression [35]. Concerning the immunological mechanisms, certain pro-inflammatory cytokines, mainly IL-6, IL-1β and TNFα, have been recently pointed out as pivotal molecules for depression-related behaviors following infection. These cytokines are likely able to coordinate local and systemic inflammatory responses to pathogens [2,22]. In the depression-like behavioral paradigm employed herein, we demonstrate that administration of LPS (450 μg/kg, i.p.) in mice previously submitted to a 5-min swimming session, produces earlier signals of sickness behavior, such as decreased body temperature and locomotor activity 6 h post-LPS, which are followed by a later manifestation of depression-like behavior, i.e. an increase of immobility time in the tail suspension test, 24 h post-LPS. This is in line with literature data indicating that sickness behavior represents a normal initial response to infectious stimuli [33,36], although depression-like states might persist after initial sickness alterations have resolved [3,37].

To the best of our knowledge, there is no previous study correlating kinin B_1 _receptors and depression. These receptors are commonly absent under normal conditions in the periphery, but might be rapidly up-regulated by stressful stimuli. Interestingly, basal expression of B_1 _receptor has been described in spinal cord and some brain structures, such as cerebral cortex, hippocampus, thalamus, hypothalamus, amygdala, and choroid plexus epithelial cells [10,38,39]. Even though the exact role of kinins in the brain is not clear, basal expression of B_1 _receptor in the nervous system is compellingly suggestive of a central role for these molecules [10,19,40]. Herein, we have assessed whether B_1 _receptors might be implicated in the depression-like behavior induced by LPS and pre-stressful stimuli.

The depression-like state observed in the tail suspension test was significantly reduced by the antidepressant imipramine, in a dose which was effective in stressed rodents [27]. Interestingly, the increased immobility was significantly inhibited by treatment with either selective B_1 _receptor antagonists R-715 or SSR240612. These results are indicative of a relevant role for B_1 _receptors in depression-like behavior. Our *in vivo *data were reinforced by real-time PCR experiments, which demonstrated a marked increase in B_1 _receptor mRNA expression in hippocampus, and notably, in the cortex of mice submitted to forced swimming plus LPS administration. Alterations of hippocampal plasticity have been widely demonstrated in depression associated with stressful insults [32,40-42]. Nevertheless, the present study is, to the best of our knowledge, the first demonstration that infection associated with a stressful stimulus might up-regulate B_1 _receptors in the CNS, leading to depressive behavior. Conversely, treatment of mice with the selective B_2 _receptor antagonist FR173657 failed to significantly affect immobility time in our depression paradigm. This is well aligned with the proposed physiological roles of kinin B_2 _receptors [43]. Considering this paradigm, the participation of B_2 _receptors was not further considered in the present study.

Of high interest, functional data obtained in the tail suspension test was extended by results employing the anhedonia paradigm of sucrose intake, in which SSR240612 was able to completely reverse reduced sucrose consumption in LPS-treated mice. It is well known that depression is likely associated with anhedonia states, where pleasure sensations are reduced or even missing [29,44]. It is noteworthy that stressful forced swimming plus LPS administration resulted in a significant decrease in sucrose consumption in Swiss mice, a parameter that was reversed by the antidepressant imipramine, which reinforces the validity of our experimental design [45]. On the other hand, sickness behavior-induced hypothermia was not significantly altered by the administration of the B_1 _receptor antagonist R-715. This evidence indicates that blocking B_1 _receptors is able to reverse later depression-like symptoms without interfering with early sickness behavior, which seems to be coordinated mainly by cytokines [46].

The major immunocompetent components of the CNS are represented by microglia and astrocytes, and the activation of these cells likely contributes to the pathogenesis of depression [43,47,48]. Our data clearly demonstrate that forced swimming followed by LPS treatment results in activation of microglia in mice, as indicated by an enhancement of CD-68 immunostaining in hippocampus 24 h after LPS injection, when B_1 _mRNA levels return to basal. It has been demonstrated that microglial activation in response to several kinds of injury drives the release of neurotoxic mediators, such as pro-inflammatory cytokines [3,49]. Of relevance, systemic treatment with the selective non-peptide B_1 _receptor antagonist SSR240612 virtually abolished the CD-68 immunopositivity, but notably this parameter was not significantly altered by imipramine. This allows us to suggest that antidepressant-like effects of B_1 _receptor antagonists, but not that caused by imipramine, are related to inhibition of microglial activation. These findings are quite relevant and open a new avenue to understand depressive states, especially those associated to infection.

Depression is clearly associated with synaptic plasticity changes, resulting in decreased BDNF function, among other biochemical alterations. It has been demonstrated that under stressful conditions, the BDNF gene is repressed leading to neuronal apoptosis in hippocampus [40,50]. Of interest, BDNF infusion in some brain regions induces antidepressant-like effects in animal models [50-52]. Additionally, most monoaminergic antidepressant drugs are known to restore normal BDNF transcriptional levels [33-35,34]. Our depression model caused a sustained reduction in BDNF mRNA expression in hippocampus (up to 24 h). Of note, BDNF mRNA expression was not significantly altered by previous administration of the selective B_1 _receptor antagonist SSR240612. Therefore, although B_1 _receptor antagonism prevented microglial activation, it failed to restore decreased brain levels of BDNF. These results suggest that SSR240612 seems to have an antidepressant-like activity by acting through different mechanisms from usual antidepressants; acting preferentially by an inflammatory pathway, and not by interfering with BDNF.

As mentioned above, pro-inflammatory cytokines are deeply involved in the pathogenesis of depression [53,54]. Of note, patients with major depressive disorder have increased expression of soluble TNFα receptors [55,56], and patients under treatment with TNFα antagonists display a general improvement of depressive symptoms and life quality [57,58]. Herein, we reinforce this hypothesis by demonstrating that LPS administration in pre-stressed mice results in a marked increase of TNFα levels in serum, CSF and whole brain. TNFα production was initially augmented in serum (between 1 and 3 h), and later increased in whole brain (between 3 and 6 h). Therefore, it is possible to argue that increased TNFα levels in serum are the main factor responsible for sickness behavioral changes (i.e. hypothermia and reduced locomotor activity), whereas the elevation of this cytokine in brain underlies the depression-like behavior in the tail suspension test. In addition, multiplex cytokine analysis demonstrated a marked increase of TNFα levels in CSF obtained from LPS-treated mice (between 1 and 3 h), although the levels of other cytokines remained unaltered. The presence of TNFα in the CSF supports the idea of a communication pathway between the brain and the immune system. When toll-like receptors on macrophage-like cells residing in the circumventricular receptors are activated, they respond by producing pro-inflammatory cytokines, such as TNFα [3]. As the circumventricular organs lie outside the blood-brain barrier (BBB), and the BBB is relatively impermeable to cytokines, the mechanisms by which circulating cytokines might influence the brain function remain a matter of debate [34]. One established theory is that they can enter the brain by volume diffusion [3,58,58]. Cytokines might additionally gain access to the brain through sites where BBB is somewhat compromised, e.g. by stressful events or immunologic challenges, such as those used in our protocol [34]. Besides, kinins enhance permeability of BBB. This would disturb BBB functioning, leading to various neuropathological conditions related to cytokine entrance into the brain, including depression [59,60].

To gain further insights into the relevance of TNFα in our experimental paradigm, we employed TNFα p55 receptor-KO mice. Both depression-like behavior and up-regulation of B_1 _receptors induced by forced swimming plus LPS treatment were virtually abolished in TNFα p55 receptor-KO mice. The relevance of TNFα for the up-regulation of B_1 _receptors has been described before by our group [14-17]. The experimental evidence provided herein clearly suggests a link between the cytokine TNFα and the kinin B_1 _receptor upregulation in depression genesis. The present data provide good evidence suggesting that generation of this pro-inflammatory cytokine seems to exert a critical role for LPS to induce B_1 _receptor upregulation, as already demonstrated [14].

Most experts agree that depression should be viewed as a syndrome, not a disease [60]. The current hypothesis linking depression and the immune system suggests that cytokines and other immune mediators work as ''sensor'' molecules, capable of transforming noncognitive stimuli (i.e. inflammatory process) into cognitive stimuli, allowing the CNS to recognize them, and to elaborate an integrated response to the peripheral event. Therefore, molecules released during peripheral inflammatory events may influence central factors controlling homeostasis and behavior [58,59]. Our findings bring a new piece of evidence into the depression disorder puzzle which may help to understand its etiology: kinin B_1 _receptors might exert a critical role in affective disorders, such as depression. The participation of B_1 _receptors in depressive alterations seems to be related to microglial activation, an event that seems to be associated with the subsequent production of TNFα in the brain. It is tempting to suggest that a clinical trial with orally available selective B_1 _receptor antagonists should be performed to evaluate whether or not these molecules could help treating clinical symptoms of depression.

## Abbreviations

BBB: blood-brain barrier; CBA: Cytometric Bead Array; DAB: 3,3'-diaminobenzidine; DG: dentate girus; HPA: hypothalamus-pituitary-adrenal; IFN-γ: interferon-γ; IL-10: interleukin-10; IL-12p70: interleukin-12p70; IL-6: interleukin-6; KO: knockout; LPS: lipopolysaccharide; PBS: phosphate buffered saline; PE: phycoerythrin; MFI: mean fluorescence intensity; TLR: Toll-Like Receptors; TNFα: tumor necrosis factor alpha.

## Competing interests

The authors declare that they have no competing interests.

## Authors' contributions

AFV; ISM; FND; CPF; JMS and MMC performed the experiments and analyzed the data. MMC and JBC participated in design and coordination, provided useful advice, wrote and reviewed the manuscript. All authors read and approved the final manuscript.
